# Gonadotropin dose selection for repeat IVF cycles in POSEIDON Groups 1 and 2

**DOI:** 10.3389/fendo.2025.1591743

**Published:** 2025-07-17

**Authors:** Hao Wei, JinLiang Duan, SiShi Wang, BaoPing Zhu, HaiLing Jiang

**Affiliations:** Reproductive Medical Center, The 924th Hospital of the Joint Logistic Support Force of the Chinese People's Liberation Army, Guilin, China

**Keywords:** *in vitro* fertilization, POSEIDON, antagonist protocol, gonadotropin dosage, cumulative live birth rate

## Abstract

**Purpose:**

Investigating whether increasing the dose of gonadotropins (Gn) in the second *in vitro* fertilization (IVF) cycle using the antagonist protocol could improve the cumulative live birth rate (CLBR) in POSEIDON Groups 1 and 2.

**Methods:**

This retrospective study included 343 patients from POSEIDON Groups 1 and 2 who underwent two consecutive cycles of ovarian stimulation with an antagonist protocol between May 2018 and September 2022. Patients were divided into an Additive group (those who increased the Gn dosage in the second cycle) and a Control group (those who maintained or decreased the Gn dosage), with a 1:2 propensity score matching analysis. The primary outcome was the CLBR.

**Results:**

In the second IVF cycle, the Additive group had higher initial (191.8 vs 183.4, P=0.135) and total (2161.7 vs 1770.6, P=0.461) Gn doses compared to the Control group. The Additive group also had a higher average number of retrieved oocytes and Metaphase II (MII) oocytes, a higher two pronuclei (2PN) fertilization rate (3.3 vs 2.6, P=0.065), and higher blastocyst formation rates (44.9% vs 44.2%, P=0.937) compared to the Control group; however, these differences were not statistically significant. The Control group had a slightly higher CLBR (31.5% vs 28.9%, P=0.8), which was also not statistically significant.

**Conclusions:**

For POSEIDON Groups 1 and 2, increasing the dose of Gn under the antagonist protocol increased treatment costs but did not improve the CLBR. Routine increase of Gn dose was not recommended.

## Introduction

The management of low-prognosis patients is a significant challenge in the field of *in vitro* fertilization (IVF). To better identify and manage POR, reproductive medicine experts introduced the Bologna criteria in 2011 (1). Subsequently, in 2016, to address the limitations of the Bologna criteria, the POSEIDON criteria were proposed (2). The POSEIDON criteria categorize low-prognosis patients into an “expected low-prognosis group” and an “unexpected low-prognosis group” based on age, antral follicle count (AFC), and anti-Müllerian hormone (AMH). The “unexpected POR group” includes Group 1, aged under 35 years, and Group 2, aged 35 years and above. A study by Esteves, S. C. et al. on 13,146 infertile women undergoing conventional ovarian stimulation showed that 43% of patients met the POSEIDON criteria, with 44% belonging to Group 1 and 36% to Group 2 (3). The unexpected low response in POSEIDON Groups 1 and 2 leads to a significant psychological gap for patients, which in turn increases the pressure on reproductive physicians in treatment. The issue of how to optimize ovarian stimulation protocols to improve treatment outcomes in subsequent IVF cycles remains controversial.

In ovarian stimulation for general IVF patients, increasing the dose of gonadotropins (Gn) is an effective means to increase the number of oocytes retrieved, and a higher initial gonadotropin dose is an independent protective factor against suboptimal response (4, 5). However, the therapeutic effect of increasing the Gn dose on POSEIDON Groups 1 and 2 currently lacks high-quality research evidence support. Existing studies lack consideration of individual variation factors in POSEIDON Groups 1 and 2 patients, and the assessment of the effectiveness of interventions for POSEIDON Groups 1 and 2 should include before-and-after comparisons in consecutive IVF cycles to exclude the interference of individual variation factors. In addition, the cumulative live birth rate (CLBR), as an important indicator for evaluating the effectiveness of IVF treatment (6), should be included in the scope of research outcomes. In the retrospective study by Parimala Chinta et al., increasing the dose of gonadotropins or changing the protocol can improve the live birth outcomes of POSEIDON Groups 1 and 2 patients (7). In the study by Alyssa Hochberg et al., through multivariate logistic regression analysis, it was found that in women with AMH values between 1.20 and 2.97 ng/mL and/or AFC between 5 and 12, increasing the dose of follicle-stimulating hormone (FSH) did not reduce the risk of suboptimal response (8). After analyzing 658,519 fresh autologous IVF cycles, Baker, V. L. et al. found that for patients with normal ovarian response, the live birth rate significantly decreased with the increase of FSH dose, a trend unrelated to the number of oocytes retrieved (9). For POSEIDON Groups 1 and 2 patients, the effectiveness of increasing the Gn dose in improving patients’ cumulative live birth rate still needs to be verified.

Therefore, we conducted a retrospective cohort study to perform a before-and-after comparison in two consecutive IVF cycles for patients in POSEIDON Groups 1 and 2, assessing the impact of increased Gn dosage during ovarian stimulation with the antagonist protocol on the CLBR of these groups.

## Study design and population

This was a single-center, retrospective cohort study targeting Poseidon Groups 1 and 2, with participants from the southern province of China. The study included cycles of utilizing sperm that was either fresh or cryopreserved. The study period from May 2018 to September 2022, and included 343 patients from POSEIDON Groups 1 and 2 who underwent repeated IVF cycles using the antagonist protocol, grouped based on the change in Gn dosage during the second IVF cycle. The Additive group consisted of 109 patients, in whom the Gn dosage was increased relative to the first IVF cycle in the second cycle. The Control group included 234 patients, in whom the Gn dosage remained stable or decreased relative to the first IVF cycle in the second cycle. The retrospective study was approved by the hospital ethics committee, and patient treatment information was obtained from the electronic medical records system.

The inclusion criteria were as follows: 1. AFC≥5 and AMH≥1.2 ng/ml; 3. patients who underwent two ovarian stimulations using the antagonist protocol within a 6-month period. The exclusion criteria included: 1. severe uterine anomalies; 2. moderate to severe intrauterine adhesions; 3. adenomyosis and endometriosis; 4. women who had oocytes cryopreserved.

All patients’ AMH tests were conducted prior to the initiation of their IVF cycles. The AFC assessment was determined on the day of ovarian stimulation initiation using two-dimensional transvaginal ultrasound, with the AFC assessment criteria referring to the practical guide published in January 2018 (10). The study’s clinical procedures were in accordance with the prevailing clinical guidelines. Ovarian stimulation for all participants commenced on day 2 through day 5 of the menstrual cycle. The dosage of the antagonist protocol was determined based on factors such as the patient’s age, BMI, and AFC. The administration of GnRH antagonists was conducted according to either a fixed or flexible protocol. Triggering was considered when the mean diameter of a follicle exceeded 18 mm or when the mean diameters of two follicles exceeded 17 mm. The preferred method for triggering was using human chorionic gonadotropin (hCG); for patients at high risk of OHSS, gonadotropin-releasing hormone agonist (GnRH-a) was used for triggering. On the trigger day, blood was drawn to measure progesterone levels to assess suitability for fresh embryo transfer (ET). Oocyte retrieval was conducted via transvaginal ultrasound-guided follicular aspiration within 36 to 38 hours following the trigger.

### Laboratory procedures

Following oocyte retrieval, the decision to use IVF or ICSI was based on sperm quality. After fertilization, embryos were individually cultured in medium with an oil overlay. All embryo culture dishes were placed in traditional incubators maintained at 37°C with an atmosphere of 5% oxygen (O_2_), 6% carbon dioxide (CO_2_), and 89% nitrogen (N_2_). Pronuclear morphology was observed 16 to 18 hours after fertilization, and embryos were evaluated daily until the day of transfer or cryopreservation. Throughout the study period, our laboratory procedures and the reagents and consumables used remained consistent.

The assessment of embryo quality was based on a scoring system that evaluated the embryos’ morphological characteristics (11). Day 3 (D3) embryos were classified into Grades I to IV based on the number and uniformity of blastomeres, and degree of fragmentation (12). Among them, Grade I and Grade II embryos were considered high-quality embryos. The Gardner grading system was employed to grade the blastocysts (13). Embryos graded as CA, CB, and CC were not subjected to transfer or cryopreservation.

For patients with multiple embryos, the sequence of embryo transfer was ascertained in accordance with the embryonic grading. For D3 embryos with identical scores, those with high pronuclei scores were prioritized for transfer. Regarding the order of blastocyst transfer, fully expanded blastocysts were prioritized over expanding ones, and the inner cell mass grade was more important than the trophectoderm grade; when the blastocysts scores were the same, the D3 embryo score was taken into account.

Fresh embryo transfers were conducted on D3 or D5 following oocytes retrieval. If fresh cycle embryo transfer was not performed, vitrification technology was used to cryopreserve the embryos. The vitrification cryoprotectant solution was primarily composed of 0.6M sucrose, 15% ethylene glycol, and 15% dimethyl sulfoxide. The principal causes for canceling fresh cycle embryo transfer encompassed the absence of transferable embryos on Day 5, preimplantation genetic testing (PGT) and OHSS. The frozen D3 embryos were not thawed for blastocyst culture.

### Thawed embryo transfer

The endometrial preparation protocols for frozen embryo transfer (FET) encompassed the natural cycle and hormone replacement therapy (HRT). The natural cycle protocol mandated that patients have regular menstrual cycles. In the hormone replacement therapy, patients orally administered 2-6mg of estradiol daily and monitored the endometrial thickness via transvaginal ultrasound; when the thickness surpassed 8 millimeters and/or after 14 days of estradiol administration, endometrial transformation was induced with an intramuscular injection of 60mg of progesterone. In our IVF center, hormone replacement therapy was typically utilized to prepare the endometrium. The day of ovulation or the day of progesterone administration was designated as Day 0, with embryo transfer conducted on Day 3 or Day 5, and the thawing and revival of the embryos accomplished on the day of transfer.

Under the guidance of transabdominal ultrasound, the embryo transfer was performed using a transfer catheter from COOK company. No assisted hatching was applied to the embryos. The number of embryos transferred was determined based on factors such as the woman’s age and embryo quality. Typically, two Day 3 embryos or one high-quality blastocyst were transferred; when patients lacked high quality blastocysts, two average-quality blastocysts might have been transferred, with a maximum of two embryos per transfer.

### Luteal support

There were mainly two approaches to post-transfer luteal support: 1. Micronized progesterone vaginal suppositories, Crinone^®^ 8% at a dosage of 90 milligrams (Merck Serono); 2. Progesterone injection solution administered via daily intramuscular injection at a dosage of 40 to 60 milligrams. Luteal support was initiated on the day of oocyte retrieval or the day of endometrial transformation. A β-hCG test was conducted on the 14th day after transfer; if embryo implantation occurred, progesterone administration would continue until 6 weeks post-transfer.

### Outcomes

The primary outcome of the study was the CLBR over two IVF cycles, which included the live birth outcomes from both fresh embryo transfers and FETs. The secondary outcomes were comprised of the clinical pregnancy rate, implantation rate, multiple pregnancy rate, pregnancy loss rate, and ectopic pregnancy rate. The criterion for clinical pregnancy was the sonographic visualization of a gestational sac. Pregnancy loss referred to the failure of a clinical pregnancy, excluding ectopic pregnancy. The live birth was defined by the presence of a heartbeat, respiration, and muscle movement in the newborn at the time of delivery. The CLBR was defined as the sum of live birth occurrences from both fresh transfers and frozen embryo transfers, with multiple births counted as one. The follow-up endpoints of the study included two scenarios: follow-up was terminated if the patient achieved a live birth; if no live birth was achieved, follow-up was terminated by November 30, 2024. From the date of the last oocyte retrieval, all patients who did not achieve live birth were followed up for over 26 months.

### Statistical analysis

The propensity score matching (PSM) was performed using the “MatchIt” package in R software. The PSM model included several covariates such as age, obstetric history, basal bFSH, AFC, dose of gonadotropin, interval between two ovarian stimulations, number of metaphase II (MII) oocytes and high-quality Day 3 embryos to estimate propensity scores. Propensity scores for each subject were estimated using logistic regression. A 1:2 nearest neighbor matching method was applied to pair each subject in the Additive group with control subjects who had the closest propensity scores. The matching process used a caliper of 0.05 to restrict the maximum distance between matching pairs, ensuring balance of covariates within specific subgroups by limiting the maximum PS difference between the matched pairs.

The distribution characteristics of the data were assessed using the Kolmogorov-Smirnov test, and continuous variables were described using the mean ± standard deviation. Normally distributed data were compared using the student’s t-test, while non-normally distributed data were compared using the Mann-Whitney U test. Categorical data were presented as frequency counts and percentage distributions, with intergroup comparisons assessed via chi-square testing. Statistical significance was set at a P-value < 0.05. The R 4.3.3 software was utilized for all statistical analyses.

## Results

### Cohort characteristics

**Figure 1:**
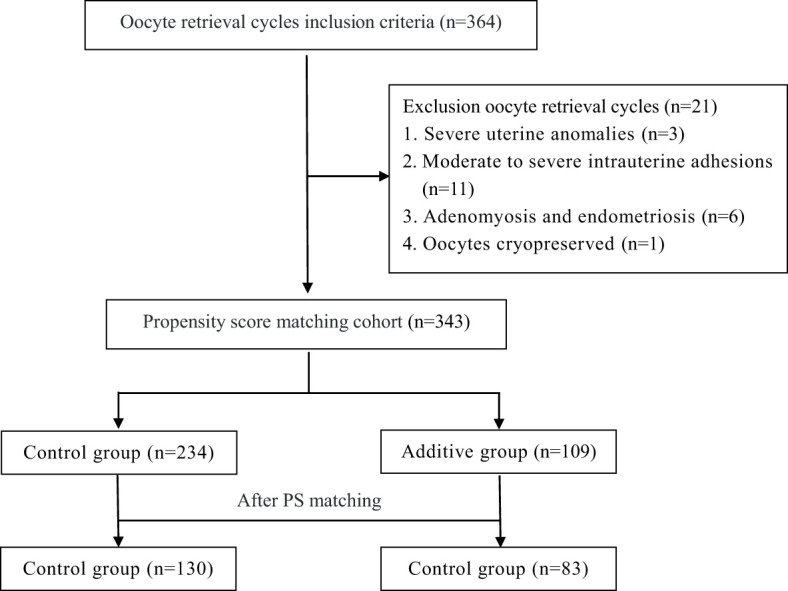
Flow chart showing the selection of the study cohort. Additive group: increased the Gn dosage in the second cycle, Control group: maintained or decreased the Gn dosage in the second cycle.

The screening process of this retrospective study cohort is shown in **Figure 1**. Initially, 364 patients from POSEIDON Groups 1 and 2 were included, and subsequently, 21 patients were excluded due to uterine anomalies, intrauterine adhesions, oocyte cryopreservation, and other reasons (**Figure 1**). Based on the inclusion and exclusion criteria of the cohort study, a total of 343 patients were enrolled. **Table 1** summarizes the demographic characteristics of the cohort before and after propensity score (PS) matching. Prior to PS matching, the Control group had significantly higher AMH and AFC compared to the Additive group [2.8 (± 2.0) vs. 2.4 (± 1.1), P=0.008] and [12.6 (± 4.9) vs. 11.6 (± 5.1), P=0.026], respectively. Apart from these differences, no statistical differences were found in baseline data between the two groups, including mean age, duration of infertility, BMI, FSH, AMH, and age of male partners. After PS matching, 130 women in the Control group and 83 in the Additive group were included in the subsequent comparative analysis; no statistically differences were found in baseline indicators such as female age, infertility duration, BMI, FSH, AMH, and AFC between the two groups (**Table 1**).

**Table 1:** Demographic and baseline IVF characteristics for PS matching.

Baseline metrics	Before PS matching			After PS matching		
	Control group	Additive group	P-value	Control group	Additive group	P-value
	(N =234)	(N =109)		(N =130)	(N =83)	
**Female age, yr** Mean (SD)	34.5 ( ± 4.5)	33.9 ( ± 4.1)	0.213	34.3 ( ± 4.5)	34.2 ( ± 4.2)	0.879
**Male age, yr** Mean (SD)	36.0 ( ± 5.4)	35.2 ( ± 5.2)	0.163	36.0 ( ± 5.5)	35.3 ( ± 5.2)	0.391
**Infertility duration, yr** Mean (SD)	4.6 ( ± 4.3)	4.2 ( ± 3.6)	0.801	4.7 ( ± 4.2)	4.0 ( ± 3.4)	0.388
**Female BMI, kg/m2** Mean (SD)	22.5 ( ± 4.3)	21.6 ( ± 3.9)	0.066	21.7 ( ± 3.3)	21.8 ( ± 4.0)	0.824
**Primary infertility,** (%)	29.1% (68/234)	35.7% (39/109)	0.260	34.6% (45/130)	36.1% (30/83)	0.819
**FSH, U/L** Mean (SD)	6.3 ( ± 2.2)	6.7 ( ± 2.4)	0.182	6.6 ( ± 2.3)	6.4 ( ± 2.3)	0.574
**AMH, ng/ml** Mean (SD)	2.8 ( ± 2.0)	2.4 ( ± 1.1)	0.008	2.6 ( ± 1.7)	2.4 ( ± 1.1)	0.427
**AFC** Mean (SD)	12.6 ( ± 4.9)	11.6 ( ± 5.1)	0.026	12.1 ( ± 4.1)	11.6 ( ± 4.8)	0.175

BMI, body mass index; FSH, Follicle stimulating hormone; AMH, anti-Müllerian hormone; AFC, antral follicle count. P<0.05 indicates a statistical difference between the two groups.

### First IVF cycle

In the first IVF cycle, the Control group had a higher initial dose of gonadotropins (Gn) and a higher total gonadotropins dose compared to the Additive group (1770.5 vs 1708.7, P=0.461), but these differences were not significant. The endometrial thickness on the trigger day was slightly lower in the Control group (10.6 vs 10.9, P=0.311), and the average number of MII oocytes was slightly higher in the Control group (5.1 vs 4.8, P=0.324). The proportion of ICSI used was also slightly higher in the Control group (30.0% vs 28.9%, P=0.871). Ultimately, the Control group had a slightly higher rate of 2PN fertilization and the number of good quality embryos on day 3 compared to the Additive group, but these differences were not statistically significant. Similarly, the blastocyst formation rate was slightly higher in the Control group (35.3% vs 32.8%, P=0.624) (**Table 2**).

**Table 2:** Demographic and baseline IVF characteristics after PS matching.

Therapeutic parameters	First IVF cycle		
	Control group	Additive group	P-value
	(N =130)	(N =83)	
**Initial dose, IU Mean (SD)**	191.8 ( ± 40.6)	183.4 ( ± 39.3)	0.135
**Total gonadotropins dose, IU,** Mean (SD)	1770.5 ( ± 577)	1708.7 ( ± 605)	0.461
**HCGEm**	10.6 ( ± 2.3)	10.9 ( ± 2.5)	0.311
**Oocytes retrieved** Mean (SD)	5.6 ( ± 2.2)	5.4 ( ± 2.1)	0.399
**MII oocytes** Mean (SD)	5.1 ( ± 2.3)	4.8 ( ± 2.2)	0.324
**ICSI cycles,** (%)	30.0% (39/130)	28.9% (24/83)	0.871
**2PN fertilization** Mean (SD)	3.2 ( ± 2.1)	3.0 ( ± 1.9)	0.644
**D3 grade I/II embryos** Mean (SD)	1.6 ( ± 1.7)	1.5 ( ± 1.6)	0.826
**Blastocyst formation rate,** (%)	35.3% (119/337)	32.8% (65/198)	0.624
Therapeutic parameters	Second IVF cycle		
	Control group	Additive group	P-value
	(N =130)	(N =83)	
**Time interval, Day** Mean (SD)	93.4 ( ± 38.6)	95.1 ( ± 40.2)	0.859
**Initial dose, IU** Mean (SD)	188.6 ( ± 43.5)	223.2 ( ± 42.2)	<0.001
**Total gonadotropins dose, IU,** Mean (SD)	1770.6 ( ± 571)	2161.7 ( ± 756)	<0.001
**HCGEm**	10.1 ( ± 2.3)	10.7 ( ± 2.3)	0.080
**Oocytes retrieved** Mean (SD)	4.5 ( ± 3.7)	4.8 ( ± 3.7)	0.614
**MII oocytes** Mean (SD)	4.1 ( ± 3.5)	4.4 ( ± 3.3)	0.380
**ICSI cycles,** (%)	39.2% (51/130)	31.3% (26/83)	0.246
**2PN fertilization** Mean (SD)	2.6 ( ± 2.3)	3.3 ( ± 2.7)	0.065
**D3 grade I/II embryos** Mean (SD)	1.4 ( ± 1.7)	1.7 ( ± 1.7)	0.111
**Blastocyst formation rate,** (%)	44.2% (140/317)	44.9% (101/225)	0.937

HCGEm, Endometrial thickness on HCG day; MII oocytes, Metaphase II oocytes; 2PN, two pronuclei; ICSI, intracytoplasmic sperm injection; P<0.05 indicates a statistical difference between the two groups.

### Second IVF cycle

In the second ovarian stimulation cycle, there was no statistically significant difference in the interval since the last ovulation stimulation between the two groups (93.4 vs 95.1, P=0.859). The Additive group had a higher initial dose of gonadotropins (Gn) compared to the Control group (191.8 vs 183.4, P=0.135), and a higher total gonadotropins dose (2161.7 vs 1770.6, P=0.461), as well as a thicker endometrial lining on the trigger day (10.6 vs 10.9, P=0.311), but these differences were not significant. The Additive group had a higher average number of retrieved oocytes (4.8 vs 4.5, P=0.614) and MII oocytes (4.4 vs 4.1, P=0.380) compared to Control group, but these differences were also not significant. The Control group had a higher proportion of ICSI fertilization compared to the Additive group (39.2% vs 31.3%, P=0.115). The Additive group had a higher 2PN fertilization rate (3.3 vs 2.6, P=0.065) and a higher number of good quality embryos on day 3 (1.7 vs 1.4, P=0.111) compared to the Control group, but these differences were not significant. Ultimately, the blastocyst formation rates were similar between the Additive group and the Control group (44.9% vs 44.2%, P=0.937), and both were higher than the blastocyst formation rates in the first IVF cycle (**Table 2**).

### Fresh embryo transfer outcomes

In the first IVF cycle, neither group had any live births following fresh ET. During the second IVF cycle, the Control group underwent 57 fresh embryo transfers, while the Additive group had 41. The average number of embryos transferred per patient was not significantly different between the two groups (1.8 vs 1.4, P=0.401). The live birth rate after fresh ET in the Additive group was lower than that in the Control group (35.1% vs 24.4%, P=0.362) (**Table 3**).

**Table 3:** Clinical outcomes after PS matching.

Pregnancy outcomes	Fresh embryo transfers in second IVF cycles		
	Control group	Additive group	P-value
	(N =130)	(N =83)G1	
**Fresh embryo transfers cycles**	57	41	
**No. of transferred embryos** Mean(SD)	1.8( ± 0.4)	1.8( ± 0.4)	0.401
**Live birth rate**, (%)	35.1%(20/57)	24.4%(10/41)	0.362
Reasons for canceling the embryo transfer			
No available embryos, (%)	31.5%(41/130)	24.1%(20/83)	0.310
PGT, (%)	7.7%(10/130)	8.4%(7/83)	0.846
OHSS, (%)	2.3%(3/130)	4.8%(4/83)	0.435
Other factors, (%)	14.6%(19/130)	13.3%(11/83)	0.792
Pregnancy outcomes	Total FET cycles		
	Control group	Additive group	P-value
	(N =130)	(N =83)G1	
**FET cycles**	91	54	
**FET Female age, yr** Mean(SD)	34.3( ± 4.8)	34.0( ± 4.1)	0.648
**HRT cycles**, (%)	73.6%(67/91)	79.6%(43/54)	0.538
**Endometrial thickness, mm** Mean(SD)	9.9( ± 2.1)	10.2( ± 2.1)	0.499
**No. of frozen embryos transferred** Mean(SD)	1.4( ± 0.5)	1.4( ± 0.5)	0.971
**Clinical pregnancy rate**, (%)	36.3%(33/91)	38.9%(21/54)	0.753
**Multiple pregnancies rate**, (%)	5.5%(5/91)	9.3%(5/54)	0.387
**Pregnancy loss rate**, (%)	17.6%(6/34)	23.8%(5/21)	0.579
**Ectopic pregnancy rate**, (%)	3.3%(3/91)	1.9%(1/54)	0.608
**Ongoing pregnancy**, (%)	3.3%(3/91)	1.9%(1/54)	0.651
**Live birth rate per FET**, (%)	23.1%(21/91)	25.9%(14/54)	0.699
**Cumulative Live birth rate**, (%)	31.5%(41/130)	28.9%(24/83)	0.800

PGT, Preimplantation genetic testing; OHSS, ovarian hyperstimulation syndrome; FET, Frozen embryo transfer; HRT, hormone replacement therapy; CLBR, cumulative live birth rate. P<0.05 indicates a statistical difference between the two groups.

### Frozen embryo transfer outcomes

In the comparison of frozen embryo transfer cycles, the Additive group underwent 91 embryo transfers, while the Control group had 54. Both groups primarily used hormone replacement therapy (HRT) for endometrial preparation, with no statistically significant difference. There was no statistical difference in the endometrial thickness before transfer or in the average number of embryos transferred. The clinical pregnancy rates between the Additive group and the Control group were not statistically different (36.3% vs 38.9%, P=0.753). The Control group had a higher rate of multiple pregnancies (9.3% vs 5.5%, P=0.387) and pregnancy loss (23.8% vs 5.5%, P=0.387) compared to the Additive group, but these differences were not statistically significant. The rates of ectopic pregnancy were also not statistically different between the two groups (3.3% vs 1.9%, P=0.651). The live birth rate following frozen embryo transfer in the Additive group was slightly lower than that in the Control group (23.1% vs 25.9%, P=0.699), without statistical significance. As of the study’s follow-up endpoint, a total of four patients in both groups were in ongoing pregnancies (**Table 3**).

### Cumulative live birth rates

In the comparison of CLBR within two IVF cycles, the Control group had a slightly higher rate than the Additive group (31.5% vs. 28.9%, P=0.8), but this difference was not statistically significant.

## Discussion

This study aimed to assess the impact of increased Gn dosage in the antagonist protocol on the cumulative live birth rate for patients in POSEIDON Groups 1 and 2. To reduce confounding factors, the study conducted a before-and-after comparison analysis of treatment outcomes in two consecutive IVF cycles. All enrolled patients initiated their second IVF cycle only after the failure of the first IVF attempt. Therefore, the CLBR in this study may have been lower than that of the general IVF population.

Ovarian reserve in women of reproductive age declines with age, and the time interval between two IVF cycles can affect treatment outcomes. Previous studies have included intervals of over one year or even several years between two IVF cycles, during which a longer interval can lead to a decrease in ovarian response due to aging (14–16). To minimize the impact of age on study outcomes, our retrospective analysis excluded cases where the time interval between two oocyte retrievals exceeded six months. Furthermore, to control for the influence of different ovarian stimulation protocols, the study only included patients who underwent both cycles with an antagonist protocol. After screening, a total of 343 infertile women were included. Following PS matching of baseline data, 130 patients in the Additive group and 83 in the Control group were analyzed. There were no statistically significant differences in baseline data between the two groups (**Table 1**). In the second IVF cycle, the initiating and total Gn doses in the Additive group were significantly higher than those in the Control group; however, there were no significant differences in the average number of retrieved oocytes, MII oocytes, 2PN fertilization, and blastocyst formation rate between the two groups. In the before-and-after comparison between the first and second IVF cycles, the number of retrieved oocytes and MII oocytes relatively decreased in the second IVF cycle, while the blastocyst formation rate and the number of blastocysts relatively increased after culture, the exact reasons for which are not yet fully understood. A possible explanation is that in the first and second groups of the POSEIDON classification, the follicles themselves have reduced sensitivity to FSH, and the number of follicles that respond to FSH is limited (17, 18), thus increasing the dose of Gn did not increase the number of retrieved oocytes and MII oocytes. However, the proportion of patients using ICSI fertilization in the second IVF cycle increased, which may be one of the reasons for the increased blastocyst formation rate and number of blastocysts. Finally, the antral follicles of the patients were recruited in a Follicular Waves pattern (19); the quality of the antral follicles recruited in the second IVF cycle may be superior to that in the first IVF cycle (20).

Eppsteiner EE et al. reported that in women with normal ovarian reserve undergoing repeated IVF cycles, increasing the gonadotropin dose was the only method that significantly increased oocyte yield (21). Studies by Out H. J and Drakopoulos P. et al. indicated that increasing the Gn dose in subsequent IVF cycles could result in a higher number of retrieved oocytes, regardless of whether the women had a normal ovarian response or a history of poor ovarian response (5, 22). In the second IVF cycle of our study, the group with increased Gn dose also obtained more MII oocytes and 2PN fertilized embryos. However, neither the studies by Drakopoulos P. nor Eppsteiner EE et al. tracked the CLBR, thus it remains unclear whether the increased oocyte yield could improve the CLBR (21, 22). Additionally, increasing the Gn dose poses additional risks to patients: excessive exogenous gonadotropins may increase the risk of OHSS (23, 24), potentially raise the incidence of embryo mosaicism (25, 26), and affect the endometrial receptivity in the fresh embryo transfer cycle of patients (27, 28); ultimately, the cumulative live birth rate of patients did not improve (29, 30).

In our retrospective study, the initiating dose and total dose of Gn in the Additive group were significantly higher than those in the Control group (P < 0.001), and the risk of moderate to severe OHSS was also higher in the Additive group. However, there was no statistically significant difference in the CLBR between the two groups. At the end of the follow-up period, 1 case in the Additive group and 3 cases in the Control group were still pregnant; however, regardless of the pregnancy outcomes, these did not affect the conclusions of the study. Therefore, increasing the dose of gonadotropin without indication not only increases the treatment cost for patients but also does not align with the modern IVF technology’s treatment philosophy of patient-friendliness and safety.

### Limitations

The data for this retrospective study were sourced from a single *in vitro* fertilization (IVF) center. The study had limited male data collection. Although PS matching was used to control for some confounding factors and inconsistencies in treatment (31, 32), potential selection bias in patient ovarian stimulation might have affected the study outcomes and limited the applicability of our findings.

## Conclusions

A comparative analysis of consecutive two IVF cycles in patients of POSEIDON Groups 1 and 2 indicated that increasing the dose of Gn under the antagonist protocol did not improve the cumulative live birth rate. Considering that increasing the dose of Gn would increase the treatment cost for patients and bring additional risks, it was not recommended to routinely increase the dose of Gn in subsequent IVF cycles for patients in POSEIDON Groups 1 and 2.

## Data Availability

The original contributions presented in the study are included in the article/Supplementary Material. Further inquiries can be directed to the corresponding author.
